# Water productivity of rainfed maize and wheat: A local to global perspective

**DOI:** 10.1016/j.agrformet.2018.05.019

**Published:** 2018-09-15

**Authors:** Juan I. Rattalino Edreira, Nicolas Guilpart, Victor Sadras, Kenneth G. Cassman, Martin K. van Ittersum, René L.M. Schils, Patricio Grassini

**Affiliations:** a Department of Agronomy and Horticulture, University of Nebraska-Lincoln, Lincoln, NE, 68583-0915, USA; b AgroParisTech, UMR Agronomie AgroParisTech INRA Université Paris-Saclay, F-78850, Thiverval-Grignon, France; c South Australian Research and Development Institute, Australia; d Plant Production Systems Group, Wageningen University, P.O. Box 430, 6700 AK, Wageningen, The Netherlands

**Keywords:** CZ(s), climate zone(s), Es:ETw, proportion of ETw evaporated from the soil during the crop cycle, ETw, seasonal water-limited potential crop evapotranspiration (mm), ETw_POSTF_ETw, proportion of ETw after flowering, ETo, reference grass-based evapotranspiration during the crop cycle (mm), VPD, daytime vapor pressure deficit (kPa), WP, water productivity (kg ha^−1^ mm^-1^), WPa, actual on-farm water productivity (kg ha^−1^ mm^-1^), WPg, water productivity gap (kg ha^−1^ mm^-1^), WPw, water-limited potential water productivity for rainfed crops (kg ha^−1^ mm^-1^), Ya, actual on-farm yield (Mg ha^-1^), Yw, water-limited yield potential (Mg ha^-1^), Water productivity, Yield, Wheat, Maize, Management, Spatial framework

## Abstract

Water productivity (WP) is a robust benchmark for crop production in relation to available water supply across spatial scales. Quantifying water-limited potential (WPw) and actual on-farm (WPa) WP to estimate WP gaps is an essential first step to identify the most sensitive factors influencing production capacity with limited water supply. This study combines local weather, soil, and agronomic data, and crop modeling in a spatial framework to determine WPw and WPa at local and regional levels for rainfed cropping systems in 17 (maize) and 18 (wheat) major grain-producing countries representing a wide range of cropping systems, from intensive, high-yield maize in north America and wheat in west Europe to low-input, low-yield maize systems in sub-Saharan Africa and south Asia. WP was calculated as the quotient of either water-limited yield potential or actual yield, and simulated crop evapotranspiration. Estimated WPw upper limits compared well with maximum WP reported for field-grown crops. However, there was large WPw variation across regions with different climate and soil (CV = 29% for maize and 27% for wheat), which cautions against the use of generic WPw benchmarks and highlights the need for region-specific WPw. Differences in simulated evaporative demand, crop evapotranspiration after flowering, soil evaporation, and intensity of water stress around flowering collectively explained two thirds of the variation in WPw. Average WP gaps were 13 (maize) and 10 (wheat) kg ha^−1^ mm^−1^, equivalent to about half of their respective WPw. We found that non-water related factors (*i.e.*, management deficiencies, biotic and abiotic stresses, and their interactions) constrained yield more than water supply in *ca.* half of the regions. These findings highlight the opportunity to produce more food with same amount of water, provided limiting factors other than water supply can be identified and alleviated with improved management practices. Our study provides a consistent protocol for estimating WP at local to regional scale, which can be used to understand WP gaps and their mitigation.

## Highlights

• Rainfed maize and wheat water productivity (WP) was assessed at local to regional scale.
• Water-limited potential WP varied across regions with different climate and soil.
• Average WP gap was 47% (maize) and 51% (wheat) of potential WP across regions.
• Variation in potential WP across regions warns against use of fixed WP benchmarks.
• Non-water related factors were usually more limiting for yield than water supply.

## Graphical abstract

**Figure:**
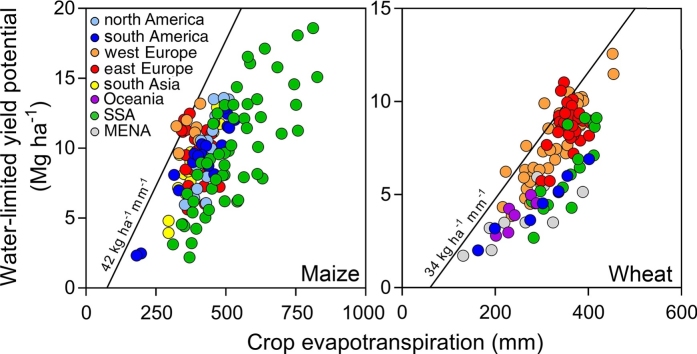

## 1 Introduction

Rising demand for food, livestock feed, and biofuels will increase competition for water resources and put pressure to improve water productivity (WP), broadly defined as the amount of agricultural output per unit of water depleted by the crop (Global Water Partnership, 2000; Rosegrant et al., 2009). Working definitions of WP require an explicit description of the numerator and denominator and the time scale (Sinclair et al., 1984; Tanner and Sinclair, 1983). From an agronomic perspective, we favor a seasonal time scale. For each definition of yield, namely potential^1^ (Yp), water-limited^2^ (Yw), and actual on-farm (Ya) yield there is a corresponding WP (WPp, WPw, and WPa). For rainfed crops, Yw and WPw are the relevant benchmarks. The denominator of the WPw equation can be crop transpiration, evapotranspiration, or water supply. The latter includes crop available soil water at sowing and in-season rainfall. WPa is typically below WPw as reported for maize and soybean in USA (Grassini et al., 2009b, 2011, 2015a), maize in China (Zhang et al., 2014), wheat in Australia, USA, China, and the Mediterranean basin (Cornish and Murray, 1989; French and Schultz, 1984; Patrignani et al., 2014; Sadras and Angus, 2006), sunflower in Argentina (Grassini et al., 2009a), and millet in sub-Saharan Africa (Sadras et al., 2011). The difference between WPw and WPa is termed water productivity gap (WPg). Robust estimates of WPw and WPg can help farmers, researchers, and policy makers estimate realistic goals of agricultural production considering available water resources and assist to identify non-water related factors that constrain WPa (Passioura, 2006; Passioura and Angus, 2010).

Previous studies that estimated WPw and WPa can be roughly grouped into two categories. The first group includes local field observations, which typically include yield, some measure of crop water availability during the crop-growing season, and a generalized boundary function representing WPw (French and Schultz, 1984; Grassini et al., 2009b; Passioura, 2006; Sadras and Angus, 2006). Recognized limitations of the boundary function approach include lack of consideration of spatial and seasonal variation in daytime vapor pressure and rainfall, and variation in soil evaporation with soil type and rainfall pattern (Angus and Van Herwaarden, 2001; Connor et al., 1985); there are also inconsistent use of crop water availability indicators (*e.g.*, seasonal water supply *versus* in-season rainfall) among studies that constrains boundary function comparisons. The second group includes regional or global studies that follow a “top down” approach to estimate WPa based on soil water balance, crop modelling, and/or remote sensing (Bastiaanssen and Steduto, 2017; Fader et al., 2011; Liu et al., 2007; Mekonnen and Hoekstra, 2010; Zwart et al., 2010). Owing to large data requirements, this approach mostly relies on gridded weather data and coarse assumptions about the crop system context, including crop sequence, management practices (sowing time and crop length), and soil water content at sowing (Fader et al., 2011; Jägermeyr et al., 2016; Mekonnen and Hoekstra, 2010; Mekonnen and Hoekstra, 2014). Perhaps more importantly, the focus of these studies is on estimating WPa, without providing a measure of WPw that can be taken as a benchmark to assess WP in farmer fields and identify opportunities for improvement.

To our knowledge, there is no protocol for estimating WPw and WPa with local to global relevance that is applicable across biophysically and agronomically diverse cropping environments. We argue that such a protocol requires (i) an accurate description of the local cropping system context (*e.g.*, weather, soil, crop sequence, and sowing dates), (ii) a robust spatial framework to upscale WPw from local to regional level, and (iii) a tool to reliably estimate Yw and the water that is available for crop transpiration during the growing season. To fill this gap of knowledge, the present study describes the protocol developed by the Global Yield Gap Atlas (Grassini et al., 2015b; van Bussel et al., 2015; www.yieldgap.org) to estimate WPw and WPa. This method is based on a combination of (i) soil, weather, and crop management data, (ii) a bottom-up approach to upscale results from location to region, and (iii) robust crop simulation models that have been validated for their ability to estimate Yw and WPw. This protocol was used to estimate WPw and WPa of rainfed crops in 17 countries for maize and 18 countries for wheat (available at www.yieldgap.org). Estimates of WPw were evaluated against data from the literature and spatial variation in WPw and WPa was investigated. Speciﬁc objectives were to evaluate the proposed approach for its ability to: (i) benchmark WPw at local and region scale across environments with contrasting climate and soil, (ii) assess drivers for WPw variation across environments, and (iii) estimate WP gaps and understand their underlying causes to gain insight into opportunities to close them.

## 2 Methods

### 2.1 Study region, site selection, and upscaling method

**Fig. 1:**
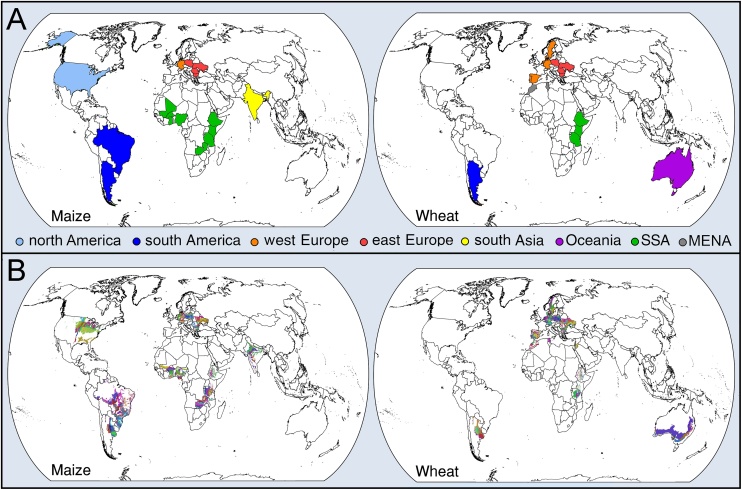
(A) Evaluated countries for rainfed maize (n_countries_ = 17) and wheat (n = 18), which represent 57 and 23% of global harvested area during the 2010–2014 period, respectively. (B) Selected climate zones for maize in north America (n_climate zones_ = 18), south America (20), sub-Saharan Africa (SSA, 54), west Europe (7), east Europe (24), and south Asia (17) and for wheat in south America (8), SSA (18), Middle East and North Africa (MENA, 10), west Europe (19), east Europe (31), and Oceania (7). Note that the color scheme to identify geographic regions in panel (A) is identical in all figures.

Maize and wheat, the most important rainfed food crops in the world, were evaluated in 17 (maize) and 18 (wheat) countries included in the Global Yield Gap Atlas (Fig. 1A), which account for 57% of global rainfed maize and 23% of global rainfed wheat harvested areas (SPAM2005 v2.0; You et al., 2014). Site selection for each country and crop was based on the protocol described by van Bussel et al. (2015) seeking to achieve a minimum of 50% coverage of national harvested crop area. Briefly, this protocol builds on the spatial framework developed by van Wart et al. (2013), which consists of delineating agro-climatic zones (CZs) based on three climate variables that influence crop yield and its variability: growing degree days, temperature seasonality, and aridity index. Within each country, CZs with >5% of total national harvested area for each crop were selected (Fig. 1B). Within each CZ, 100-km radius buffer zones (*ca.* 7800 km^2^) were created and "clipped" by CZ boundaries to ensure that each buffer zone was located within a unique CZ. Buffer zones were sequentially selected based on their contribution to national crop harvested area until *ca.* 50% national crop area coverage was achieved. If needed, additional buffers were added to include regions with high crop area density but without a weather station. In our set of 26 countries, there were 245 (maize) and 196 (wheat) buffer zones, in a total of 140 (maize) and 112 (wheat) CZs, which, in aggregate, accounted for 80% (maize) and 85% (wheat) of the national harvested areas. Details on site selection method and evaluation of the approach can be found elsewhere (Hochman et al., 2016; van Bussel et al., 2015; van Wart et al., 2013). To simplify visual presentation of the results, only aggregated data at CZ or (sub-) continental levels are shown.

### 2.2 Protocol to estimate water-limited potential and actual water productivity

Water-limited yield potential (Yw) and seasonal evapotranspiration (ETw) were estimated for rainfed crops using simulation models that fulfill the criteria by van Ittersum et al. (2013). Briefly, we favored models that fit these criteria: (i) daily step simulation, (ii) crop specificity, (iii) flexibility to simulate key management practices like sowing date, plant density, and cultivar maturity, (iv) simulation of key physiological processes including crop development, net carbon assimilation, biomass partitioning, crop water relations, and grain growth, (v) low requirement of genetic coefﬁcients, (vi) validation against data from ﬁeld crops that approach Yp and Yw, (vii) user friendly, and (viii) full documentation of model parametrization and parameters availability. Simulation models calculate daily ETw between sowing and physiological maturity, which can be aggregated to derive seasonal ETw. Daily ETw is simulated based on evaporative demand, soil water content, and crop leaf area. Daily changes in soil water content are computed based on precipitation, ETw, and water losses through surface runoff and deep drainage. ETw represents the amount of water that is available for transpiration during the growing season by a crop without nutrient limitations and free of biotic adversities. Agronomically, our simulated ETw is the crop water supply required to achieve Yw, accounting for the temporal distribution of water supply and after discounting unavoidable water losses through runoff and deep percolation, and residual available soil water at physiological maturity.

Water-limited potential water productivity (WPw) was calculated as the quotient of Yw and ETw. To conform to the definition of Yw, simulations of yield and ETw assumed no nutrient deficiency, pathogens, pests, and weeds, and no extreme stresses such as heat and waterlogging. Instead of using a single model globally, models were selected for each particular region based on their ability to reproduce locally measured yield in well-managed wheat and maize (van Ittersum et al., 2013). Maize simulations were performed with (i) Hybrid-Maize in USA, Brazil, India, and sub-Saharan Africa (SSA) (Yang et al., 2017, 2004), (ii) CERES-maize in Argentina (Aramburu Merlos et al., 2015; Jones and Kiniry, 1986; Jones et al., 2003; Monzon et al., 2012), and (iii) WOFOST in Europe (Boogaard et al., 2014; van Diepen et al., 1989). For wheat simulations, we used (i) CERES-wheat in Argentina (Aramburu Merlos et al., 2015; Monzon et al., 2007; Ritchie and Otter, 1985), (ii) APSIM in Australia (Carberry et al., 2013; Hochman et al., 2009; Keating et al., 2003; McCown et al., 1996), and (iii) WOFOST in Europe, SSA, and Middle East and North Africa (MENA) (Wolf et al., 2011) (Table S1).

Simulations were based on local weather, soil, and key management practices influencing Yw, such as sowing date and cultivar maturity, which were collected following the tier-approach for selection of best available data sources described by Grassini et al. (2015b). Actual records with 10–20 years of daily weather data were available for 86% (maize) and 95% (wheat) of selected weather stations and their buffer zones. Weather data included incident solar radiation, maximum (Tmax) and minimum (Tmin) air temperature, humidity, wind speed, and precipitation. Weather data were screened for erroneous and/or missing information using rigorous quality-control protocols available at: http://www.yieldgap.org/web/guest/methods-weather-data. We followed two approaches to derive 10–20 years weather data for those buffer zones for which such long-term weather records were unavailable. In those buffers in which measured weather data were available for <3 years, we generated longer records following the propagation technique described in van Wart et al. (2015). Briefly, this technique consists of using *ca.* 3 years of location-speciﬁc measured daily weather to correct for bias in gridded Tmax and Tmin from the Prediction of Worldwide Energy Resource (NASA-POWER) dataset while missing solar radiation and precipitation data are filled using uncorrected data from NASA POWER (NASA, 2017) and Tropical Rainfall Measuring Mission (Kummerow et al., 2000) databases, respectively. In buffer zones without any weather data, we used uncorrected gridded weather data from NASA-POWER. The first approach (*i.e.*, propagation) was followed for 8% (maize) and 3% (wheat) of the buffer zones, and the second one (*i.e.*, uncorrected NASA-POWER data) for 6% (maize) and 2% (wheat) of the buffer zones.

Within each buffer zone, dominant soil type x crop sequence combinations were simulated. Yw is sensitive to soil hydraulic properties that govern plant-available water retention characteristics, and landscape and soil properties that influence infiltration rate and runoff. Soil input data used by different crop models to simulate Yw differ to some extent. However, basic soil information required by all models consists of (i) rootable soil depth, (ii) available water holding capacity (difference in water content between field capacity and permanent wilting point), either as direct input or estimated from soil texture using pedotransfer functions, and (iii) terrain slope and drainage class (for calculating surface runoff). We used high-quality soil maps with functional soil properties where these were available (*e.g.* north America, Europe, and Oceania). Otherwise, we used the global ISRIC-WISE soil databases such as ISRIC-WISE (Batjes, 2012) and AfSIS (Leenaars et al., 2018). Dominant soil types were selected to achieve >50% area coverage per buffer zone (Grassini et al., 2015b; van Bussel et al., 2015). Details on selected soil data sources can be found in http://www.yieldgap.org/web/guest/methods-soil-series. Agronomic information including crop sequences, commonly used cultivars, crop cycle length, and sowing date window were obtained from local experts. For some regions (*e.g.*, sub-Saharan Africa), sowing date was simulated dynamically for each buffer-year based on daily precipitation dynamics within the reported sowing window. For crops simulated with Ceres-Maize and APSIM, available soil water content at sowing was estimated by simulating the soil water balance during the entire crop sequence over years, including the fallow period. Lacking this option for crops simulated with Hybrid-Maize and WOFOST, the soil water balance was initiated near (or slightly after) harvest time of the preceding crop using a fixed soil water content, which was retrieved from expert opinion and/or simulation of water balance for the previous crop.

We did not attempt to estimate the actual crop evapotranspiration, which is likely to be below our simulated ETw due to non-water related constraints. For example, sub-optimal nutrient supply and root diseases can reduce crop water uptake, transpiration-use efficiency, and harvest index (*e.g.*, Angus and Van Herwaarden, 2001; Brueck 2008; Cooper et al., 1987). Instead, the goal of our study was to analyze actual on-farm yields (Ya) relative to the water availability during the crop growing season. As mentioned previously, the simulated ETw represents the amount of water that is available for transpiration during the growing season by a crop growing without nutrient limitations and free of biotic adversities, accounting for unavoidable water losses and residual available water. Hence, actual on-farm water productivity (WPa) was calculated as the ratio of Ya and ETw. If WPa ≈ WPw, it means that the crop efficiently used and converted the available water supply into grain yield. In contrast, if WPa is much lower than WPw, it means that other non-water related factors prevented the crop from fully utilizing the available water supply and converting it into grain yield. Official statistics on Ya were collected for each crop-country combination at the finest spatial resolution for which these data were available (*e.g.*, county, department, or sub-district depending upon country). Ya was determined by including as many recent years of data as possible to account for weather variability, while avoiding the trend bias due to technology or climate change (Calviño and Sadras, 2002; Grassini et al., 2015b; van Ittersum et al., 2013). In all cases, Ya was calculated with at least 3 recent years of yield data. Details on criteria for selection of data sources can be found elsewhere (Grassini et al., 2015b). Water productivity gap (WPg) was calculated as the difference between WPw and WPa and reported as a percentage of WPw.

Yield potential (Yp), Yw, and ETw were simulated for each dominant crop sequence and soil type within each buffer zone using 10–20 years of daily weather data. Simulated yields and WPw estimations were first aggregated to buffer zone level based on crop area shares of each combination of crop sequence and soil type. Subsequently, buffer zone results were upscaled to CZ, national, and (sub-)continental levels using a weighted average based on harvested area retrieved from SPAM2005 v2.0 (You et al., 2014) or better national estimates of crop areas. Details on the upscaling method can be found in van Bussel et al. (2015). Because the objective of our study was to understand variation in WPw and WPg across environments, averages of Yw, ETw, and WPa per CZ across the simulated period were evaluated. Yield and WP were expressed at 15.5% (maize) and 13.5% (wheat) grain moisture content.

### 2.3 Comparison with published data and analysis of variation across environments

We compared our estimates of WPw against boundary functions reported in the literature (Connor et al., 2011; Grassini et al., 2009b; Sadras et al., 2015). Selected boundary functions were also compared against a large database of measured data from rainfed and irrigated field-grown crops (Grassini et al., 2009b; Sadras and Angus, 2006; Zwart and Bastiaanssen, 2004 and references therein). Irrigated WP data were included to strengthen the comparison, especialy in the upper range of ETw. The boundary function was assumed to have an *x*-intercept (*i.e.*, minimum soil evaporation) of 75 mm for maize and 60 mm for wheat, and a slope (*i.e.*, transpiration-use efficiency) set at 42 kg ha^−1^ mm^−1^ for maize and 34 kg ha^−1^ mm^−1^ for wheat (Connor et al., 2011; Grassini et al., 2009b; Sadras and Angus, 2006). Slopes of boundary functions and data from the literature were also expressed at 15.5% (maize) and 13.5% (wheat) grain moisture content.

Causes for variation in WPw across CZs were investigated for each crop. Linear regression was used to reveal associations between WPw and several possible factors including (i) average reference grass-based evapotranspiration (ETo) and daytime VPD, (ii) fraction of ETw after flowering (ETw _POSTF_ : ETw), (iii) fraction of ETw evaporated from the soil (Es : ETw), and (iv) water stress index around flowering (± 10 days), which indicates the degree of crop stress due to water limitation (Cooper et al., 1983; Passioura and Angus, 2010; Steduto et al., 2012). Daily water stress index was calculated as one minus the ratio between simulated water-limited transpiration and non-water limited transpiration. Daily values were averaged for the period bracketed between ± 10 days around flowering, which corresponds to silking (maize) and anthesis (wheat); this period is critical for grain number determination in both crop species (Fischer, 1985; Hall et al., 1981). Model II regression was used to account for error in both WPw and explanatory variables (Ludbrook, 2012; Niklas, 1994). Stepwise, multiple-linear regression was used to study joint effects of multiple factors on WPw.

### 2.4 Understanding productivity gaps

To determine the degree to which water or other factors limited crop yield, we calculated two yield gaps: (i) between Yp and Yw to define a yield gap due to water, and (ii) between Yw and Ya to account for non-water related factors at given level of water supply. Non-water related factors include biotic (insect, weeds, pests, and diseases) and abiotic (frost, hail, waterlogging, heat stress) stresses, deficient management, and their interactions. We delineated three regions with equal area within the “water” *versus* “non-water related factors” gap plot to categorize CZs as (i) mostly limited by water, (ii) mostly limited by other factors, or (iii) equally limited by both. A CZ was categorized as limited by either water or other factors when one of the two gaps exceeded the other by more than 73% (*i.e.*, tan 60° or tan 30° ^−1^, which delineate the three equal-size regions) or as equally limited by both when the difference was smaller. Subsequently, we computed the percentage of crop area that corresponds to each gap category for each study region. We note that our estimates of Yp are conservative as they assumed same management (sowing date, plant density, and cultivar maturity) as for the rainfed crop, whereas Yp of fully irrigated crops can often benefit from longer growing season and higher plant population (Grassini et al., 2009b). Still, such a comparison between the yield gap due to water and other factors is useful to determine the degree to which water limits current on-farm yields *versus* other environmental stresses, management, and their interactions.

## 3 Results

### 3.1 Potential water productivity across rainfed crop producing areas

**Fig. 2:**
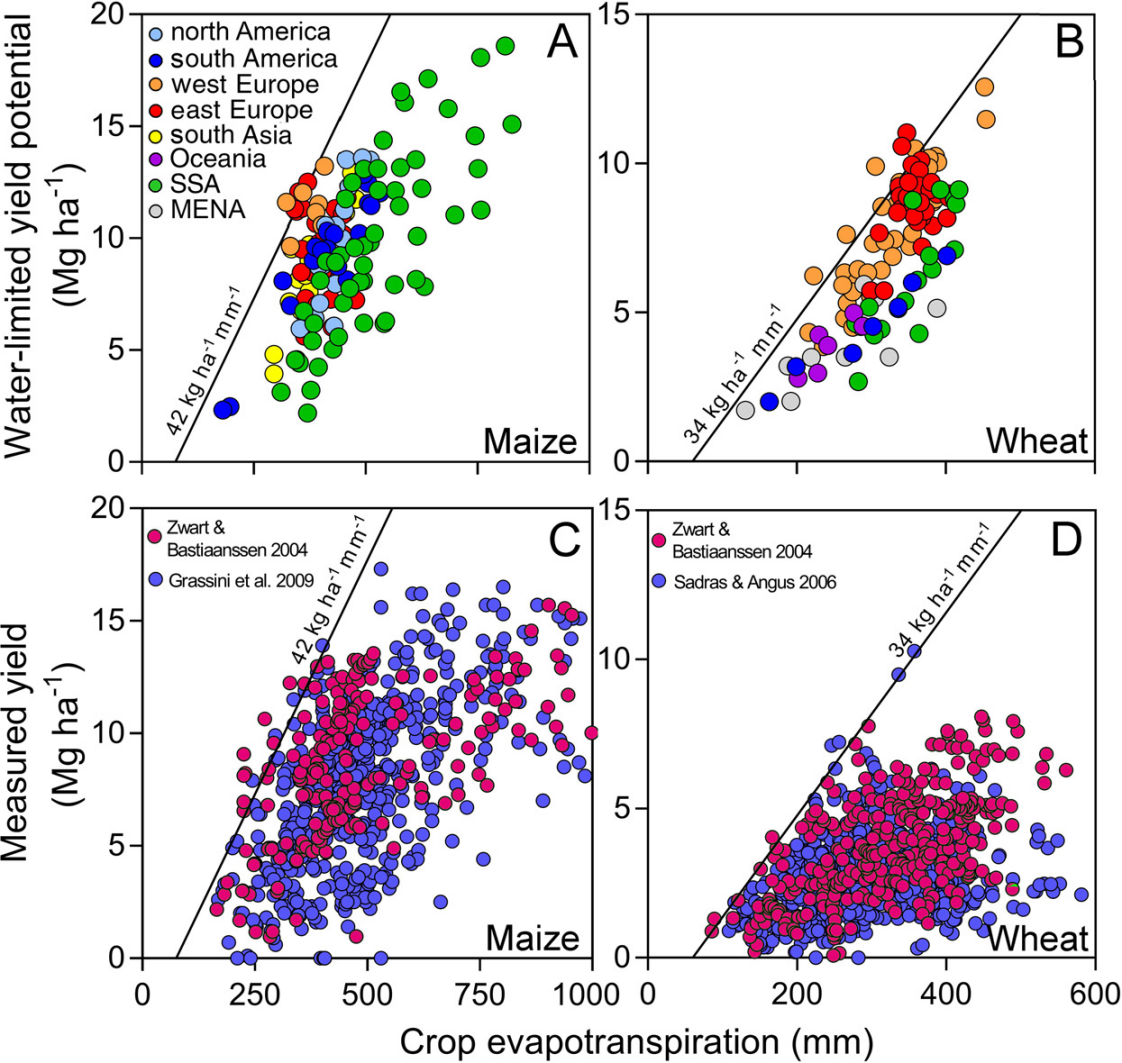
Relationship between simulated water-limited yield potential (Yw) and seasonal water-limited potential crop evapotranspiration (ETw) for (A) maize and (B) wheat across crop producing regions in North and south America, west and east Europe, sub-Saharan Africa (SSA), south Asia, Oceania, and Middle East and North Africa (MENA). Each datapoint corresponds to the 10–20 years average Yw and ETw for a given climate zone. Measured yield versus measured crop evapotranspiration for (C) maize and (D) wheat as reported by Grassini et al. (2009b); Sadras and Angus (2006); Zwart and Bastiaanssen (2004) and references therein. In all panels, solid lines are shown following the French and Schultz (1984) frontier concept, with minimum soil evaporation set at 75 mm for maize (Grassini et al., 2009b) and 60 mm for wheat (Sadras and Angus, 2006), and WPw set at 42 and 34 kg ha^−1^ mm^−1^ for maize and wheat, respectively (Connor et al., 2011). The boundary functions and data from the literature were expressed at 15.5% (maize) and 13.5% (wheat) grain moisture content.

There was large variation in both Yw and ETw across CZs reflecting the diversity of climate, soil, and cropping systems (Fig. 2). Yw ranged from 2.2 to 18.6 Mg ha^−1^ for maize and 1.3 to 12.6 Mg ha^−1^ for wheat. ETw varied from 138 (harsh environments in south America) to 810 mm (favorable regions in SSA) for maize, and from 150 mm in harsh environments in south America and MENA to 500 mm in favorable environments in west Europe for wheat. Variation in ETw was attributable not only to climate and soil, but also to the length of crop-growing season (in days) for both crops (*p* < 0.001; *r^2^* > 0.18). For instance, maize crop-growing season varied from *ca.* 3 months in Burkina Faso up to 6 months in the Ethiopian highlands.

Upper limits of Yw and ETw were linearly related over the range of water supply in which grain yield was responsive to increasing water availability, which was consistent with boundary functions from the literature (Fig. 2A, B). Those boundaries were also shown to represent the upper limit of WPa across the world based on field measurements (Fig. 2C,D; adapted from Zwart and Bastiaanssen, 2004). The wide variation in Yw at any ETw is reflected in coefficients of variation for WPw of 29% for maize and 27% for wheat. For example, Yw varied from 4 to 13 Mg ha^−1^ (maize) and 5 to 10 Mg ha^−1^ (wheat) across CZs with ETw of ≈400 mm (Fig. 2A,B). This variation in WPw warns against the use of static WPw across environments, highlighting the need to derive CZ-specific WPw. Aggregated at regional level, WPw ranged from 18 (SSA) to 29 kg ha^−1^ mm^−1^ (west Europe) for maize, and from 15 (south America) to 24 kg ha^−1^ mm^−1^ (west Europe) for wheat. Average maize and wheat WPw across all countries included in our analysis, weighted by production area in each region, was 23 and 20 kg ha^−1^ mm^−1^, respectively.

### 3.2 Drivers for variation in WPw across environments

**Fig. 3:**
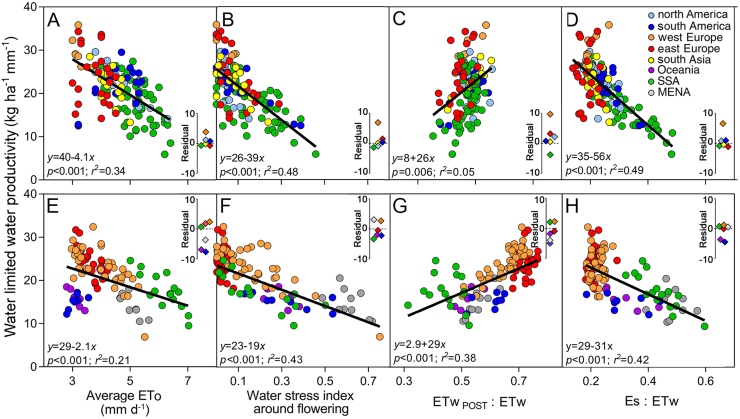
Water-limited potential water productivity for rainfed (A–D) maize and (E–H) wheat plotted against (A, E) average reference evapotranspiration (ETo), (B,F) water stress index around flowering, (C,G) proportion of seasonal water-limited potential crop evapotranspiration after flowering (ETw_POST_ : ETw), and (D,H) soil evaporation fraction during the crop cycle (Es : ETw). Each datapoint corresponds to a 10–20 year average calculated for a climate zone. Fitted lines are model II regressions that account for error in both WPw and explanatory variables. Inset: average residual from the fitted equation for each region (in kg ha^−1^ mm^−1^).

**Fig. 4:**
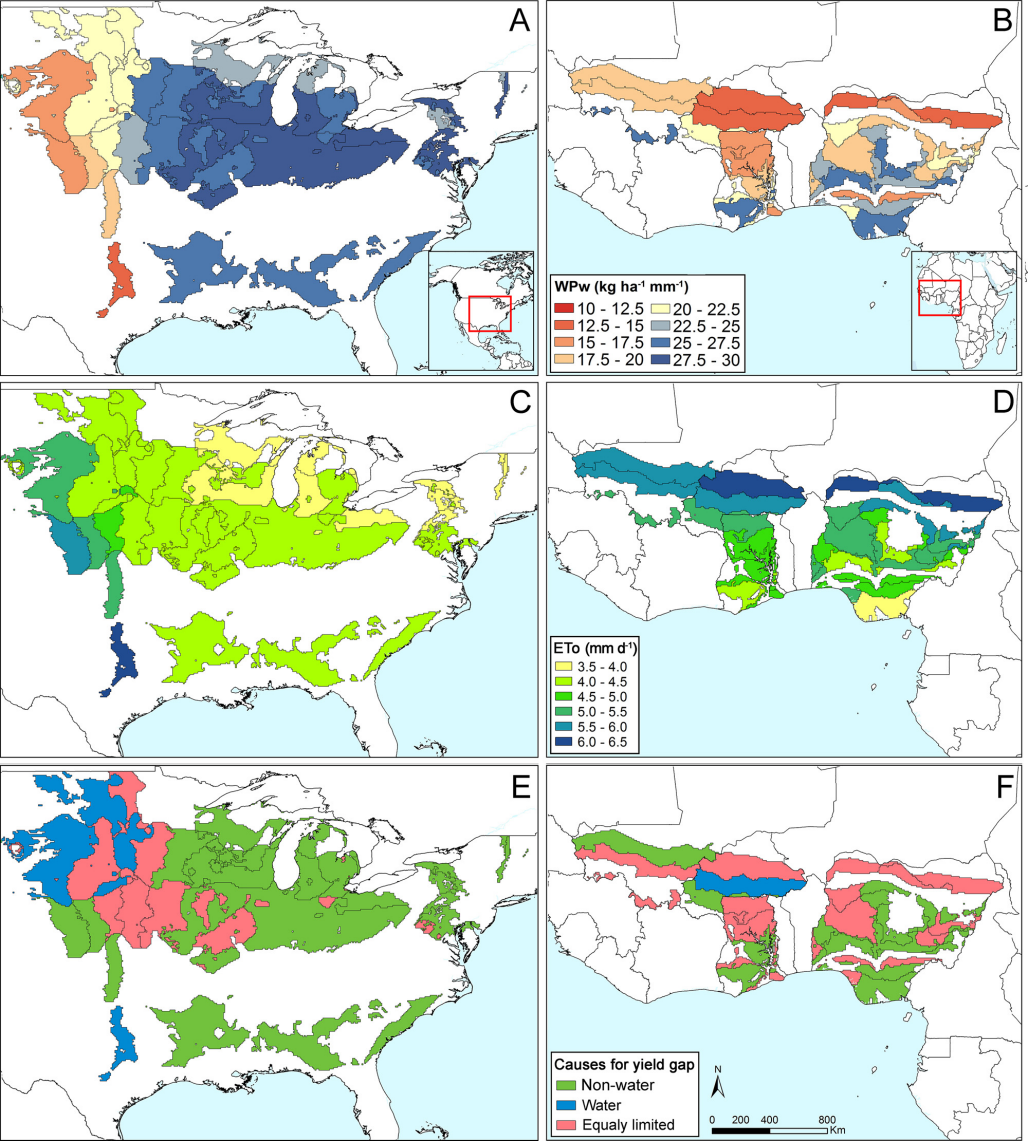
Maize water productivity and factors affecting it in two regions. (A, B) Water-limited potential water productivity (WPw), (C, D) average grass-referenced evapotranspiration (ETo), and (E, F) causes for yield gap across climate zones in north America (USA) and west Africa (Mali, Burkina Faso, Ghana, and Nigeria).

Low WPw was associated with high ETo during the crop cycle, severe water stress around flowering, small proportion of ETw after flowering, and large soil evaporation fraction (Fig. 3). For example, average maize WPw decreased from 28 to 10 kg ha^−1^ mm^−1^ with an increase of evaporative demand from 3 mm d^−1^ in Europe and the north-central US region to 7 mm d^−1^ in SSA and western US Corn Belt (Fig. 3A). Spatial variation in WPw due to variation in ETo is illustrated for maize in north America and west SSA in Fig. 4. Consistent with Steduto et al. (2007), results from our analysis based on ETo and daytime VPD were similar (data not shown), though ETo exhibited greater explanatory power. Hence, only results based on ETo are presented here (Fig. 3A). WPw decreased with increasing water deficit around flowering and fraction of soil evaporation (Fig. 3B, C). There was a positive association between WPw and the proportion of ETw after flowering (Fig. 3D). Similar trends were observed for wheat (Fig. 3E–H). Analysis of residuals indicated that the average residual from the fitted equation varied across region and crops (Fig. 3 inset). For example, for the same level of water stress around flowering, or proportion of ETw after flowering, or evaporation fraction, there was a higher maize WPw in west Europe than in other regions. In the case of wheat, the largest deviation (negative residuals) was observed for ETo and evaporation fraction in Oceania and south America. Multiple-regression models, including all the four factors in Fig. 3, explained 67% of total variance in WPw for maize and 65% for wheat (Table 1).

**Table 1:** Multiple-regression analysis for the relationship between water-limited potential water productivity and several variables including (i) average reference evapotranspiration (ETo) during the crop cycle, (ii) water stress index around flowering, (iii) proportion of seasonal water-limited potential crop evapotranspiration consumed after flowering (ETw_POST_ : ETw), and (iv) quotient between soil evaporation and evapotranspiration during the crop cycle (Es : ETw) for rainfed maize and wheat across crop producing regions: north and south America, west and east Europe, sub-Saharan Africa, south Asia, Oceania, and Middle East and North Africa. Parameter estimates and associated standard error and *t*-test are shown for statistically significant explanatory variables (p < 0.05).

Crop	Adjusted *r*^2^	Variable	Estimate ± standard error	*T* value	*P* value
Maize	0.67	Intercept	33.3 ± 2.9	11.7	<0.001
		Water stress index	−18.2 ± 3.2	−5.8	<0.001
		Es : ETw	−21.2 ± 5	−4.2	<0.001
		ETw _POST_ : ETw	15 ± 4.9	3.1	0.003
		ETo	−2.7 ± 0.4	−6.7	<0.001
Wheat	0.65	Intercept	21.9 ± 2.5	8.9	<0.001
		Water stress index	−12.6 ± 2	−6.2	<0.001
		ETw _POST_ : ETw	13.5 ± 3.9	3.5	0.007

### 3.3 Water productivity gaps

**Fig. 5:**
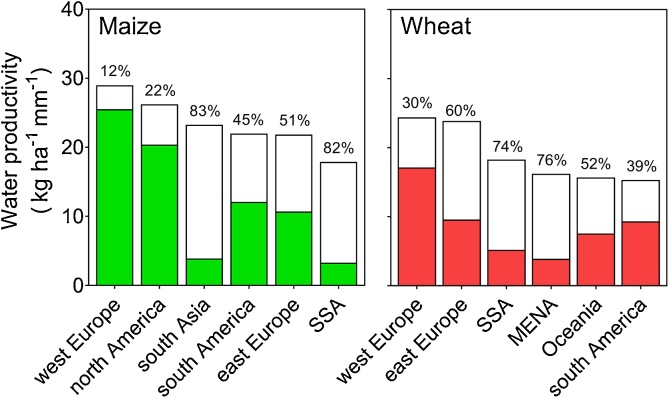
Average (10–20 years) water-limited potential water productivity for rainfed maize and wheat across producing regions: north and south America, west and east Europe, sub-Saharan Africa (SSA), south Asia, Oceania, and Middle East and North Africa (MENA). The colored portion of the bars indicates the actual water productivity and the open portion represents the water productivity gap. The water productivity gap, expressed as a percentage of WPw, is shown above bars.

There was wide variation in WPg across regions for both maize and wheat. For example, maize WPa was below 4 kg ha^−1^ mm^−1^ in south Asia and SSA, which represented a WPg of *ca.* 80% of their WPw (Fig. 5). In contrast, west Europe and north America exhibited highest maize WPa (26 and 20 kg ha^−1^ mm^−1^, respectively), which corresponded to a WPg of 12 and 22% of their WPw, respectively. Similarly, SSA and MENA exhibited low WPa for wheat (5 and 4 kg ha^−1^ mm^−1^), while west Europe showed the highest WPa (17 kg ha^−1^ mm^−1^). Across all regions, average WPg, weighted by production areas in each CZ, was 13 and 10 kg ha^−1^ mm^−1^ for maize and wheat, respectively, which represents about half of their respective average WPw.

**Fig. 6:**
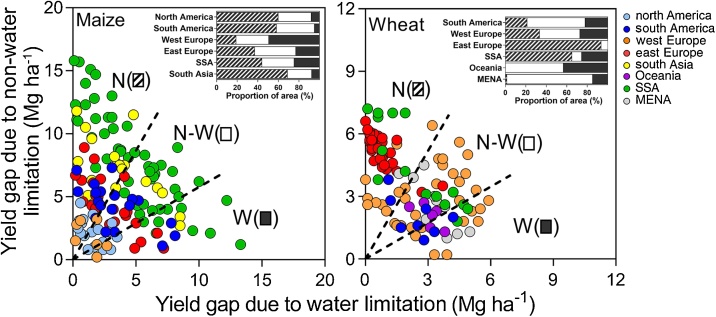
Yield gap due to non-water *versus* water limitating factors for maize and wheat in each climate zone (CZ). The yield gap due to water limitation was calculated as the difference between yield potential (Yp) and water-limited yield potential (Yw), while the yield gap due to non-water limitation was calculated as the difference between Yw and actual on-farm yield (Ya). Lines separate CZ where yields are predominantly limited by water (W), non-water related factors (N), or similarly limited by both (N-W). Insets show the fraction of cropland area within each target region that falls in each (N, N-W, W) category.

To quantify the contribution of water and other factors to yield gaps, Fig. 6 plots the difference between Yw and Ya (‘yield gap due to non-water limiting factors’) *versus* the difference between Yp and Yw (‘yield gap due to water limitations’) for each CZ. About 19, 49, and 32% of maize CZs and 18, 47, and 35% of wheat CZs were categorized as dominantly limited by water, limited by non-water limiting factors, or equally limited by both factors, respectively. In other words, we found that non-water limiting factors constrain yield substantially more than water in about half of the CZs accounted for by our study. Even in very harsh environments for rainfed crop production such as Oceania and MENA, non-water related factors were as limiting as water supply. Spatial variation in the causes for yield gap is illustrated for maize in north America and west SSA (Fig. 4E, F).

## 4 Discussion

Understanding how much grain a region can potentially produce in rainfed systems per unit of available water (*i.e.,* WPw) and how much it currently produces (*i.e*., WPa) is essential to estimate the untapped crop production potential with available water resources without irrigation. However, there has been no explicit effort to develop a generic method that ensures both local relevance and scaling to global level to estimate WPw and WPa for rainfed production areas. Our study expanded on previous studies on water productivity for specific geographic regions (*e.g.*, Doorenbos and Kassam, 1979; French and Schultz, 1984; Sadras and Angus, 2006) by developing a bottom-up, agronomically-relevant approach to determine WPw and WPg at local, regional, and national or sub-continental levels for rainfed cropping environments with diversity in climate and soil. Our study progresses previous efforts to estimate WPa (Bastiaanssen and Steduto, 2017; Zwart et al., 2010) by providing a robust estimate of potential water productivity (*i.e.*, WPw) for major crop producing regions. At local level, WPw can be used as a benchmark for current WP, estimate realistic goals of agricultural production considering available water resources, and help identify non-water limiting factors. While these WP metrics alone are not sufficient to evaluate the sustainability of cropping systems and water resources, they are essential to understand the interactions at the food-water nexus and identify trade-offs and evaluate opportunities for improvement.

The relationship between Yw and ETw reported here followed the expected positive association between these two variables (French and Schultz, 1984). The upper limits of WPw for maize and wheat were consistent with those described in the literature (Connor et al., 2011; Grassini et al., 2009a; Sadras and Angus, 2006; Zwart and Bastiaanssen, 2004), demonstrating the robustness of the proposed approach. Our approach was also able to capture variation in WPw associated with evaporative demand, fraction of ETw lost as soil evaporation, water stress around flowering, and seasonal partitioning of ETw across environments. These findings are consistent with previous reports and highlights the importance of temporal distribution of water supply during the crop-growing season (Çakir, 2004; Calviño et al., 2003; Kemanian et al., 2005; Monzon et al., 2012; Tollenaar, 1991). Large WPw variation warns against broad use of static WPw across environments with contrasting weather and further highlights the need to derive CZ-specific WPw. In absence of robust WPw estimates, the relationships reported in the present study between WPw and the aforementioned variables can be used as a first step to derive WPw for a given environment.

We acknowledge that there is uncertainty related with model ability to simulate Yw and ETw as well as with underpinning weather, soil, and crop system data. For example, previous inter-model comparisons showed variation among models in simulated Yw and ETw (Asseng et al., 2013, Camargo and Kemanian, 2016; Cammarano et al., 2016). However, model choice is an unlikely source of bias as we used models that have been explicitly evaluated for their performance to simulate Yw and/or ETc in the regions covered in our study or for similar biophysical environments (*e.g.,* Aramburu Merlos et al., 2015; Carberry et al., 2013; Hochman et al., 2009; Monzon et al., 2007; Monzon et al., 2012; van Diepen et al., 1989; Wolf et al., 2011; Yang et al., 2017). We recognize that some regions have additional sources of uncertainty due to coarse model calibration as a result of lack of high quality experimental data and scarcity of weather and soil data (Grassini et al., 2015a,b, van Wart et al., 2015). The relative contribution of these factors (model choice *versus* weather, soil data, and model calibration) to the overall uncertainty is difficult to assess. Our study calculates WPw based on best available data for these regions, recognizing that more efforts in collecting better experimental, weather, and soil data are needed to improve these estimates.

Our analysis showed a large variation in WPg among regions. As expected, regions where crops received adequate nutrient inputs and pest control (*e.g.*, west Europe and north America) had smallest WPg (Gobin et al., 2017; Mekonnen and Hoekstra, 2014). In contrast, gaps were larger (>75% of the WPw) in regions where farmers experience limitations to access inputs, markets, and extension services (*e.g.*, SSA, south Asia). The majority of the crop production environments analyzed in this study were more limited by non-water limiting factors than by water, even in dry environments, which is consistent with previous reports (Cornish and Murray, 1989). There are three causes explaining the non-water related yield gap. First, environmental factors including biotic and abiotic stresses such as frost, hail, waterlogging, heat stress and soil chemical (*e.g.*, salinity, acidity) and physical constraints (*e.g.*, compaction), which are unaccounted for the simulation of Yw (Barlow et al., 2015; Sadras et al., 2005). Second, poor management practices such as inadequate sowing date, plant density or uneven stands, inadequate fertilization, and insufficient weed, pest, and disease control, leading to reduction in farmer yields (Rattalino Edreira et al., 2017; Tokatlidis and Koutroubas, 2004). Third, interactions between environmental stresses and management; for example, reduced plant population and nitrogen input to manage risk in drought-prone areas (Grassini et al., 2014; Sadras, 2004). Overall, the findings from this study indicate that there is an important untapped food production potential with available water resources that can be exploited through tuning of current management factors. Realizing this extra potential will depend on identifying major non-water limiting factors in each region and availability of cost-effective interventions to ameliorate them without increasing farm risk (Sadras et al., 2016).

## 5 Conclusions

The approach proposed here combines local weather, soil, and agronomic data, and crop modeling in a spatial framework to determine WPw, WPa, and WPg. Maximum WPw estimated across CZs were consistent with previous studies on boundary functions based on field measurements. Notably, the approach captured variation in WPw across CZs with contrasting climate and soils, which was associated with evaporative demand, fraction of ETw lost as soil evaporation, water stress around flowering, and seasonal partitioning of ETw. Across regions, average WPg weighted by production area in each CZ was 13 (maize) and 10 (wheat) kg ha^−1^ mm^−1^, representing about half of their respective average WPw values. Non-water related factors (*i.e.*, management deficiencies, biotic and abiotic stresses, and their interactions) were more limiting for yield than water supply in *ca.* half of the CZs, which highlights the opportunity to produce more food with the same amount of water. Our study provides a consistent protocol for assessing WPw and WPa that can be used as a starting point to understand water productivity gaps and their mitigation. For example, our approach has potential to serve as basis to benchmark on-farm water productivity across fields located with the same CZs and identify cohorts of cost-effective management practices that consistently lead to high WPa given the same climate-soil context. It can also help as a tool to evaluate impact of research and extension programs aiming at increasing crop production for the same amount of water resources and, when complemented with other biophysical and socio-economic data, help guide expansion of irrigated crop production. Estimates of WPa and WPw at local and regional scale for different crops and countries are available at: www.yieldgap.org.

## Footnotes

1 Yield potential (Yp) is the yield of a crop cultivar when grown in an environment to which it is adapted, with non-limiting water and nutrient supplies, and with insect, pests, weeds, and diseases effectively controlled (Evans, 1993, van Ittersum and Rabbinge, 1997)

2 In rainfed systems where water supply from stored soil water at sowing and in-season rainfall is not enough to meet crop water requirement, water-limited yield potential (Yw) is determined by water supply amount and its distribution during the growing season, and by soil properties influencing the crop water balance, such as rootable soil depth, available-water holding capacity, and terrain slope (van Ittersum et al., 2013).
