# Supplementary material for: Water productivity of rainfed maize and wheat: A local to global perspective
Source: Agric For Meteorol. 2018 Sep 15;259:364–73. doi: 10.1016/j.agrformet.2018.05.019 (PMC6018065; doi:10.1016/j.agrformet.2018.05.019)
Supplement: Supplementary file 1 [file mmc1.docx]

**Table S1:** *Actual yield (Ya), water-limited yield potential (Yw), seasonal water-limited potential crop evapotranspiration (ETw), actual water productivity (WPa), and water-limited potential water productivity (WPw) for rainfed maize and wheat in different countries. National harvested area (and % of global area) and crop simulation model(s) used for each crop-region are shown.*

| Crop | Region | Country | 2010-2014 area | Simulation model | Ya | Yw | ETw | WPa | WPw |
| --- | --- | --- | --- | --- | --- | --- | --- | --- | --- |
|  |  |  | (Mha) |  | (Mg ha^-1^) | (Mg ha^-1^) | (mm | (kg ha^-1^ mm^-1^) | |
| Maize | sub-Saharan Africa (SSA) | Burkina Faso | 0.8 (0.4) | Hybrid Maize | 1.5 | 6.2 | 374 | 4.0 | 16.6 |
|  |  | Ethiopia | 2.0 (1.0) |  | 2.2 | 12.6 | 690 | 3.2 | 18.2 |
|  |  | Ghana | 1.0 (0.5) |  | 1.7 | 8.3 | 444 | 3.9 | 18.8 |
|  |  | Kenya | 2.1 (1.0) |  | 1.9 | 8.1 | 555 | 3.4 | 14.7 |
|  |  | Mali | 0.7 (0.3) |  | 1.9 | 9.7 | 463 | 4.1 | 20.9 |
|  |  | Nigeria | 5.4 (2.5) |  | 1.7 | 11.2 | 503 | 3.3 | 22.3 |
|  |  | Tanzania | 3.7 (1.8) |  | 1.2 | 5.3 | 436 | 2.6 | 12.0 |
|  |  | Zambia | 1.1 (0.5) |  | 2.3 | 11.3 | 573 | 4.0 | 19.8 |
|  | south Asia | India | 8.9 (4.2) | Hybrid Maize | 1.5 | 9.5 | 403 | 3.8 | 23.6 |
|  | west Europe | Germany | 0.5 (0.2) | WOFOST | 9.7 | 11.0 | 379 | 25.4 | 28.9 |
|  | east Europe | Bulgaria | 0.4 (0.2) | WOFOST | 5.9 | 7.3 | 374 | 15.7 | 19.6 |
|  |  | Poland | 0.5 (0.2) |  | 6.1 | 10.5 | 377 | 16.3 | 27.8 |
|  |  | Romania | 2.5 (1.2) |  | 3.4 | 9.0 | 438 | 7.8 | 20.6 |
|  |  | Ukraine | 4.0 (1.9) |  | 4.7 | 8.2 | 358 | 13.0 | 22.8 |
|  | north America | USA | 34.3 (16.2) | Hybrid Maize | 9.7 | 12.4 | 472 | 20.5 | 26.3 |
|  | south America | Argentina | 4.0 (1.9) | CERES-maize | 6.8 | 11.6 | 504 | 13.5 | 23.0 |
|  |  | Brazil | 14.2 (6.7) |  | 4.6 | 8.7 | 378 | 12.2 | 23.0 |
| Wheat | SSA | Ethiopia | 1.6 (0.6) | WOFOST | 1.8 | 6.7 | 353 | 5.1 | 18.9 |
|  |  | Kenya | 0.2 (0.1) |  | 2.5 | 6.1 | 367 | 6.8 | 16.5 |
|  |  | Tanzania | 0.1 (0.04) |  | 1.0 | 4.5 | 337 | 2.9 | 13.3 |
|  | west Europe | Denmark | 0.7 (0.3) | WOFOST | 7.1 | 8.1 | 370 | 19.3 | 21.9 |
|  |  | Germany | 3.2 (1.3) |  | 7.6 | 9.7 | 373 | 20.4 | 26.1 |
|  |  | Netherlands | 0.2 (0.1) |  | 8.8 | 11.6 | 454 | 19.4 | 25.5 |
|  |  | Portugal | 0.1 (0.02) |  | 1.5 | 5.4 | 256 | 6.0 | 21.0 |
|  |  | Spain | 2.1 (0.9) |  | 3.1 | 6.5 | 292 | 10.4 | 22.1 |
|  |  | Sweden | 0.4 (0.2) |  | 6.2 | 8.7 | 337 | 18.4 | 25.9 |
|  | east Europe | Poland | 2.2 (0.9) | WOFOST | 4.2 | 9.7 | 349 | 11.9 | 27.7 |
|  |  | Romania | 2.1 (0.8) |  | 3.6 | 8.4 | 366 | 9.8 | 22.8 |
|  |  | Bulgaria | 1.2 (0.5) |  | 3.6 | 8.1 | 376 | 9.7 | 21.6 |
|  |  | Ukraine | 6.2 (2.6) |  | 3.1 | 8.2 | 362 | 8.5 | 22.8 |
|  | Middle East & North Africa (MENA) | Jordan | 0.02 (0.01) | WOFOST | 1.0 | 2.8 | 232 | 4.1 | 12.2 |
|  |  | Morocco | 3.1 (1.3) |  | 1.2 | 2.6 | 181 | 6.5 | 14.6 |
|  |  | Tunisia | 0.7 (0.3) |  | 0.6 | 3.6 | 211 | 3.1 | 17.0 |
|  | Oceania | Australia | 13.3 (5.5) | APSIM | 1.7 | 3.6 | 231 | 7.5 | 15.7 |
|  | south America | Argentina | 3.8 (1.6) | CERES-wheat | 3.0 | 5.0 | 323 | 9.4 | 15.4 |
